# IoT-Based Assessment of a Driver’s Stress Level

**DOI:** 10.3390/s24175479

**Published:** 2024-08-23

**Authors:** Veronica Mattioli, Luca Davoli, Laura Belli, Sara Gambetta, Luca Carnevali, Andrea Sgoifo, Riccardo Raheli, Gianluigi Ferrari

**Affiliations:** 1 Multimedia Laboratory, Department of Engineering and Architecture, University of Parma, 43124 Parma, Italy; veronica.mattioli@unipr.it (V.M.); riccardo.raheli@unipr.it (R.R.); 2 Internet of Things (IoT) Laboratory, Department of Engineering and Architecture, University of Parma, 43124 Parma, Italy; luca.davoli@unipr.it (L.D.); laura.belli@unipr.it (L.B.); 3 Stress Physiology Laboratory, Department of Chemistry, Life Sciences and Environmental Sustainability, University of Parma, 43124 Parma, Italy; sara.gambetta@unipr.it (S.G.); luca.carnevali@unipr.it (L.C.); andrea.sgoifo@unipr.it (A.S.)

**Keywords:** Driver Monitoring System, Internet of Things, IoT, arousal, wearable, thermal camera

## Abstract

Driver Monitoring Systems (DMSs) play a key role in preventing hazardous events (e.g., road accidents) by providing prompt assistance when anomalies are detected while driving. Different factors, such as traffic and road conditions, might alter the psycho-physiological status of a driver by increasing stress and workload levels. This motivates the development of advanced monitoring architectures taking into account psycho-physiological aspects. In this work, we propose a novel *in-vehicle* Internet of Things (IoT)-oriented monitoring system to assess the stress status of the driver. In detail, the system leverages heterogeneous components and techniques to collect driver (and, possibly, vehicle) data, aiming at estimating the driver’s arousal level, i.e., their psycho-physiological response to driving tasks. In particular, a wearable sensorized bodice and a thermal camera are employed to extract physiological parameters of interest (namely, the heart rate and skin temperature of the subject), which are processed and analyzed with innovative algorithms. Finally, experimental results are obtained both in simulated and real driving scenarios, demonstrating the adaptability and efficacy of the proposed system.

## 1. Introduction

Over the last five decades, research activities focusing on the impact of driving tasks on the psycho-physiological status of a driver have gained an increasing scientific interest. Indeed, driving can be considered a major experience in many people’s life. Instances of early works on this topic mainly focus on the relationship between traffic congestion and stress [1,2,3], demonstrating that conditions of high congestion are associated with higher levels of perceived stress. However, driver stress may also be influenced by other external factors (e.g., roadway conditions, weather, visibility, etc.) and internal factors (e.g., gender, age, personality, etc.) [4]. Moreover, it has been demonstrated that the driving context (e.g, road objects, signs, vehicles, etc.) has a significant impact on the driver’s status [5]. Attempts to characterize the driving context from driver behavior have been carried out in [6], where car trajectory segmentation is exploited to derive meaningful characteristics of the driving context. Hence, both external and internal factors may negatively affect the driving performance by altering the psycho-physiological status of the driver, possibly causing road accidents and dangerous situations.

This further motivates the development of advanced monitoring systems able to extract and process physiological parameters to evaluate the psycho-physiological activation of a subject as a response to the driving task. Indeed, the perception of stressful stimuli causes a psycho-physiological reaction in the human body by altering its functions. In particular, physiological processes are regulated by the Autonomic Nervous System (ANS), which is composed of the sympathetic and parasympathetic systems [7]. A psycho-physiological activation corresponds to the activation of the sympathetic system, which regulates body functions in stressful conditions, and a simultaneous deactivation of the parasympathetic system, which regulates body functions at rest. In this work, we refer to the psycho-physiological activation of the driver with the term *arousal*.

Among different indices that might be observed, an important informative physiological index of the stress level of a subject is the heart rate variability (HRV), which can be defined as the beat-to-beat temporal fluctuation in heart rate (HR) [8]. In particular, the HRV reflects the activity of the ANS, which may be influenced by stressful stimuli and/or mental efforts. Moreover, HRV can be inferred from an ElectroCardioGram (ECG) and has been considered in various research works in the context of driver stress detection [9,10,11,12,13]. An extensive review of works where the HRV is considered as the main physiological parameter used to detect stress in different contexts can be found in [14]. This review provides evidence to support the use of the HRV for the objective assessment of physiological stress. Further examples of works, which highlight the reliability of the HRV as stress indicator, are [15,16], where it is demonstrated that mental stress can be inferred from HRV analysis.

Given the importance of the HRV as a stress indicator, several attempts at developing driver assistance systems (DASs) based on the monitoring of physiological parameters of interest have been proposed in the literature. As an example, in [17] a DAS equipped with a stress monitoring system was designed to provide assistance in electric vehicles by intervening with the vehicle speed in case of anomalies, whereas in [18], a Machine Learning (ML)-oriented stress detection system to be possibly integrated with a DAS is presented. Both systems presented in [17,18] are based on the analysis of ECG signals and other physiological data, including skin temperature (among others). Indeed, variations in the skin temperature of a subject can be considered another important stress indicator. In particular, the skin temperature on the nasal tip region is regulated by the ANS and has been observed to decrease in the case of high levels of stress [19]. Therefore, thermal imaging-based systems able to detect facial skin temperature variations have been developed in simulated driving environments [20,21] and in real driving scenarios [22]. In general, the extensive analysis carried out in the literature on the use of HRV and facial skin temperature to estimate the stress status of a subject proves how these two physiological parameters are considered significant indicators of stress by the research community.

On the basis of these remarks, in this work, the design and implementation of an advanced *in-vehicle* Driver Monitoring System (DMS) able to collect and jointly process physiological data, including HRV signals and facial skin temperature variations, to evaluate the arousal level of the driver, are presented. More in detail, the proposed DMS is composed of interconnected heterogeneous sensors properly interacting according to different Internet of Things (IoT)-oriented technologies. In particular, wireless communication protocols, such as Wi-Fi and Bluetooth Low Energy (BLE), and standard messaging protocols, such as the Message Queuing Telemetry Transport (MQTT) protocol and Transmission Control Protocol (TCP)-based sockets, are exploited to collect physiological data extracted by a wearable sensor and a thermal camera integrated in the system. Recent progress on wearable device technologies makes them effective for a wide range of applications, including those in the healthcare sector [23]. In particular, flexibility and stretchability are conferred to the most advanced wearable devices by innovative electronic materials, which make them comfortable and adaptable. To this end, recent examples of the successful development of innovative wearable sensors can be found in [24,25,26]. The effectiveness of the DMS was evaluated in both simulated and real driving scenarios according to specific operational protocols. This work expands upon previous contributions preliminarily presented in [27,28], where the first versions of the system architecture and of a thermal imaging system were introduced, respectively.

More in detail, the main purpose of this work was to demonstrate the feasibility of the developed DMS, in terms of data collection (through commercial IoT devices) considering a realistic driving scenario. More thorough comparative analyses with other existing systems are left as a future research activity, as they are out of the scope of the present study. Moreover, the proposed IoT-based architecture differentiates itself from other approaches described in the literature (e.g., [29,30]), where ML models are considered to estimate the driver’s mental workload and distractions only in simulated scenarios. It also differs from other approaches mentioned in this paper (namely, the work in [9]), since the number of employed sensors and extracted physiological signals is different. In particular, in [9], five wearable sensors were employed and needed to be attached to different parts of the subject’s body, making the system more invasive and uncomfortable than a DMS, which requires only one wearable sensor and a contactless thermal camera.

Finally, we highlight that the experimental setup in the real vehicle scenario does not actually consider the integration of Engine Control Unit (ECU) data, since access to this information is generally forbidden by manufacturers on commercial vehicles. Despite this, the proposed DMS can, in principle, be improved by including data fusion with *in-vehicle*-related information (provided that access to it is granted). This paper is organized as follows. In Section 2, the architecture of the proposed IoT monitoring system, together with a brief description of each component, is provided. The operational procedure for collecting data obtained through the involved sensors is detailed in Section 3, where the interactions between the system components are described. In Section 4, we present innovative algorithms to process and further analyze the collected data. The performance results obtained in simulated and real environments are discussed in Section 5. Finally, in Section 6, conclusions and possible future extensions are summarized.

## 2. In-Vehicle Monitoring Architecture

**Figure 1:**
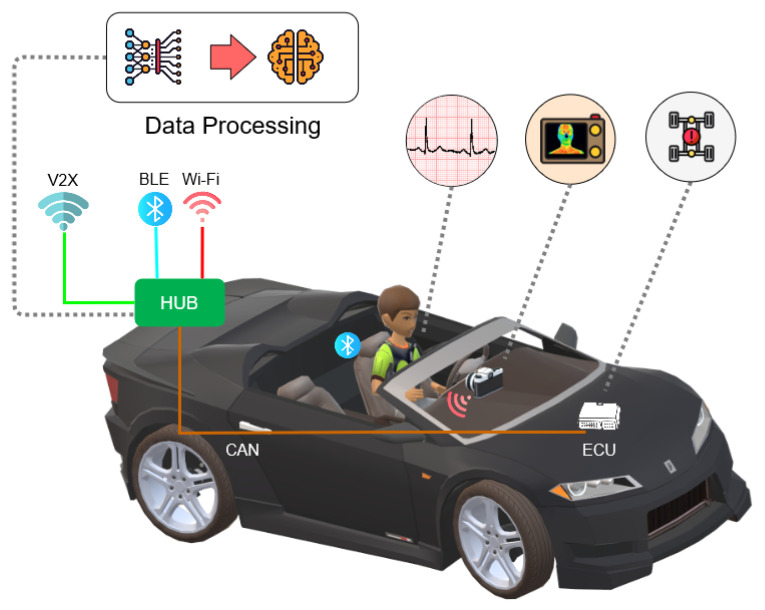
Experimental setup of the proposed DMS.

The proposed DMS is composed of heterogeneous sensing and processing devices that should properly be positioned inside the vehicle cabin and connected with each other. For the sake of clarity, the experimental system setup is shown in Figure 1, where the various components and their interactions are shown. In particular, a wearable sensor and a thermal camera are employed to gather physiological parameters of interest and record facial skin temperature variations in the driver, respectively. Additional vehicular data might be extracted by embedded inertial sensors, i.e., an ECU located on board the vehicle. The processing component in the proposed DAS is an Intel Next Unit of Computing (NUC), acting as a Wi-Fi Access Point (AP), providing a private Wi-Fi network, and also providing networking, storage and processing functionalities for the entire system.

### 2.1. Wearable Sensor

**Figure 2:**
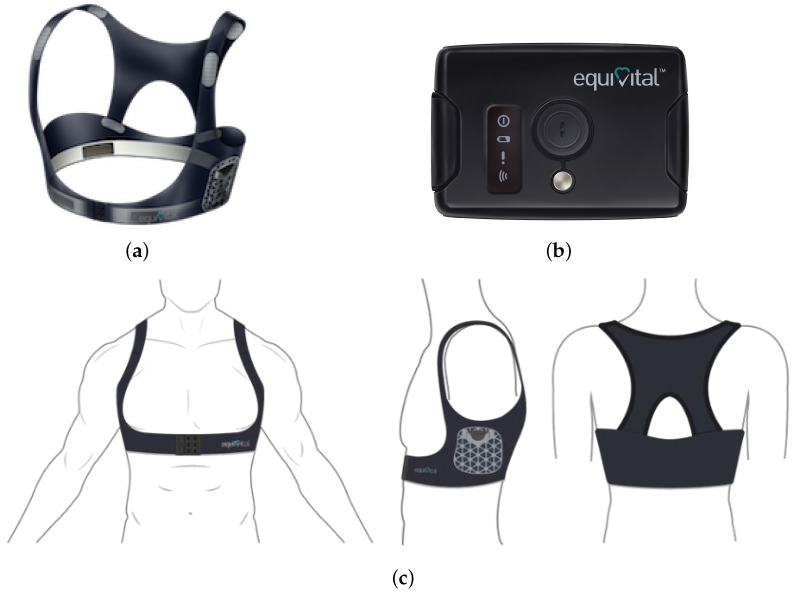
Equivital EQ02 Life Monitor sensor: (**a**) belt, (**b**) SEM, and (**c**) positioning.

In order to collect physiological data from the driver, an Equivital EQ02 Life Monitor [31] device (manufactured by Equivital, Cambridge, UK) is employed. In detail, the Equivital EQ02 Life Monitor is a wireless wearable sensing device capable of recording multiple vital signs related to a subject wearing the sensor, and is composed of two elements: (i) a textile bodice embedding electrode, i.e., the EQ02 Sensor Belt, and (ii) a EQ02 Sensor Electronics Module (SEM). The SEM can capture biological signals in real time, including ECG and respiratory signals and other indices such as HR, respiratory rate (RR) and skin temperature. Additional information on the body position and motion can be collected from a 3-axis accelerometer embedded in the SEM. Moreover, the SEM is equipped with a Class-1 Bluetooth interface for real-time data transmission. Alternatively, the acquired data may be logged on the sensor and *a posteriori* downloaded. Pictures of the EQ02 Sensor Belt and SEM are shown in Figure 2a and Figure 2b, respectively, whereas their correct on-body positioning is illustrated in Figure 2c. As can be seen, the SEM needs to be inserted in a specific pocket on the left side of the belt itself. Thus, this wearable device can be considered *non-invasive* since it is made of a flexible fabric that can easily adapt to the body of the driver. Owing to its wearing comfort, this device is also employed by athletes and workers during their regular activities, as described in [32,33].

### 2.2. Thermal Camera

The second component integrated in the architecture of the proposed monitoring system is a FLIR One Pro LT thermal camera [34] (manufactured by Teledyne FLIR LLC, Wilsonville, OR, USA), which allows us to collect thermal information from specific regions on the body of the driver (namely, the face and nose) and from the surrounding environment. This device is composed of an RGB sensor and an infrared sensor, allowing us to simultaneously capture visible and thermal images or videos, respectively. Data may also be acquired according to a blended modality, providing a combination of visible and thermal outputs.

**Figure 3:**
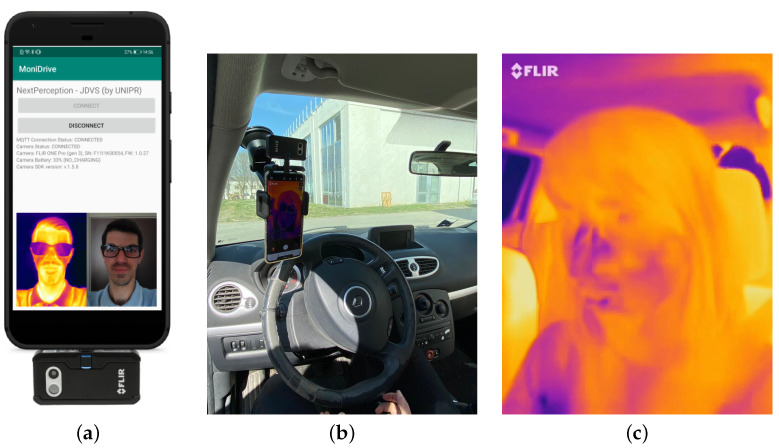
FLIR One Pro LT thermal camera: (**a**) sensor connection, (**b**) positioning, and (**c**) recorded infrared frame.

On the practical side, the FLIR One Pro LT thermal camera has to be connected to a smartphone as an external USB Type-C “dongle” and may be managed by either Android- or iOS-like mobile applications. The smartphone and connected thermal camera dongle need to be accurately installed inside the vehicle cabin to guarantee optimal positioning for data acquisition. To this end, a fair trade-off between recording quality and positioning obtrusiveness should be considered, since further video processing and analysis tasks require a frontal perspective. Then, similarly to the wearable sensor, this device can also be considered *non-invasive* since it does not require direct contact with the subject and is positioned unobtrusively, without limiting the road view. For the sake of illustration, the connection of the FLIR dongle to the smartphone and their positioning inside the cabin are shown in Figure 3a and Figure 3b, respectively. An illustrative example of a captured infrared video frame is shown in Figure 3c. Finally, we highlight that the thermal camera has small dimensions (namely, 68×34×14 mm), making it easily positionable by means of a standard phone car holder attached to the front windscreen of the vehicle, as shown in Figure 3b. Hence, thanks to this simple positioning, the thermal camera can be considered as not distracting and/or stressful for the driver.

## 3. Data Acquisition

### 3.1. Data Acquisition Architecture

**Figure 4:**
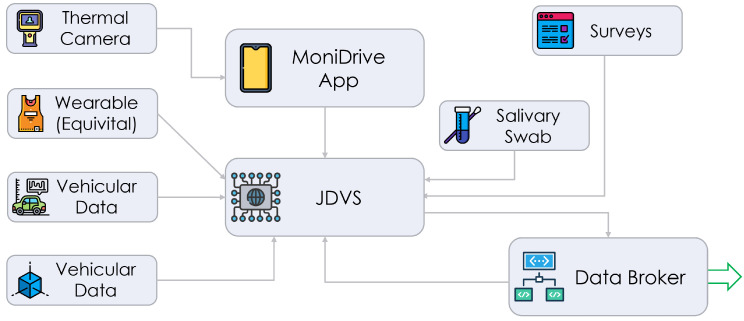
Data acquisition architecture of proposed DMS.

The different modules detailed in Section 2 and composing the proposed driver monitoring architecture transmit their collected data to the central hub, namely, the Intel NUC running Windows 10, located on board the vehicle and intended to work as a multi-interface gateway for data storage, fusion and processing. A pictorial representation of the data acquisition architecture of the proposed DMS is shown in Figure 4, where the connections between the different components are highlighted and the central hub is denoted as the Joint Driver–Vehicle Status (JDVS) module.

With regard to the interconnection of the sub-modules, standard communication protocols are employed. In particular, the EQ02 SEM sends physiological data (i.e., the HR, HRV and RR of the subject wearing the wearable bodice) through its Class-1 Bluetooth interface to the JDVS module, where the Equivital eqView Pro [35] desktop application is installed and collects the physiological data. These data are then forwarded to a TCP socket toward an internal Python application (running on board the JDVS module), hosting a TCP server and listening for incoming packets for further processing. On the other hand, thermal data recorded by the FLIR One Pro LT thermal camera are transmitted (by the smartphone hosting the FLIR dongle), exploiting the MQTT protocol, to the JDVS module through the private Wi-Fi network hosted and advertised by the JDVS itself. To this purpose, an Android-based mobile application, denoted as MoniDrive (as shown in Figure 4) and running on a Huawei P20 Lite smartphone, was developed to acquire RGB, thermal images and additional frame and camera information (i.e., temperature scale, framed hottest and coldest points, etc.) through the FLIR camera connected on its USB-C interface. Finally, serial communication channels, such as the Controller Area Network (CAN) bus, might also be exploited to transmit vehicular data from an ECU. In this study, we did not investigate this aspect. Moreover, the proposed DMS was designed to support this decision.

We remark that vehicle vibrations do not affect the data acquisition phase. In fact, the employed wearable bodice is not sensitive to small vibrations. Moreover, the proposed video processing techniques allow us to detect a framed face regardless of the camera position and orientation, with the subject’s facial temperature not being affected by vehicle vibrations in any way. As a consequence, *in-vehicle* vibrations occurring during the driving activities do not represent a noise source in the experimental data collection phase. The JDVS module asynchronously processes the data received from the various components (every 1 s from the wearable sensor and every 5 s from the thermal camera, respectively) in order to produce an arousal index (denoted as φ) in the range [0,1], where 0 and 1 indicate null and maximum physiological activations.

To summarize, the operational tasks performed by the JDVS module are the following:

- Acting as a Wi-Fi AP (advertising a private Wi-Fi network) and as a MQTT broker;
- Processing the information received from the MoniDrive app, through innovative image and video processing algorithms that will be detailed later;
- Processing the data received from the sensorized belt;
- Performing data fusion to estimate the arousal φ and transmit it to external interested entities (e.g., the vehicular ECU through a parallel Ethernet or Wi-Fi interface).

Finally, with regard to the communication protocols adopted in the proposed IoT-based monitoring architecture, it should be highlighted that, even if the use of BLE, Bluetooth and, more in general, wireless technologies in a vehicle could lead to interference problems [36,37], this issue has not been experienced in our experimental data collection campaigns. However, this aspect would deserve full attention, should the proposed system be integrated, as an *on-board* functionality, in a real vehicle.

### 3.2. Driving Protocol

With regard to a realistic deployment and the validation of the proposed data acquisition architecture introduced in the previous section, specific experimental scenarios were designed to evaluate the driver’s response to different external stimuli, associated with different amounts of perceived stress. To this end, the driving sessions monitored through the proposed system were run in both a controlled environment (namely, a driving simulator), and in realistic scenarios (namely, urban and beltway driving with smooth and heavy traffic), in order to analyze the variations in the driver’s psycho-physiological status in both smooth and fast driving conditions. As an example, in [38], an experimental route including urban and beltway driving was defined to collect and analyze data on the stress status of the driver in different driving contexts.

#### 3.2.1. Real Driving

**Figure 5:**
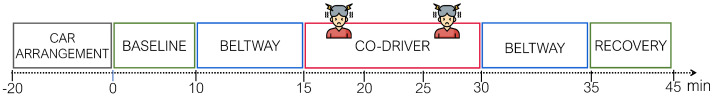
Driving protocol adopted in realistic scenarios.

In this work, a first example of a driving protocol defined for acquiring data in real driving situations is shown in Figure 5. In detail, the driving protocol consists of specific epochs—with their duration shown in Figure 5—associated with different amounts of perceived stress induced by various external factors, e.g., road type, traffic conditions and co-driving. The face icons in Figure 5 delimit the time interval when additional stress is induced on the driver by the co-driver. To this end, it is useful to highlight how, in the specific context of real driving, the considered stress is a mental stress. Indeed, the stress induced by road and traffic conditions and by the presence of a co-driver is aimed at increasing the driver’s mental workload.

Before starting each driving test, the driver and vehicle are equipped with the proposed IoT monitoring system during an initial arrangement phase. In particular, the driver is asked to wear the Equivital EQ02 Life Monitor sensor, while the FLIR One Pro LT thermal camera (attached to a smartphone) is properly positioned inside the vehicle cabin according to the guidelines provided in Section 2.1 and Section 2.2. Moreover, during this phase the stress surveys are also administrated.

The various phases of the 45 min driving tests in realistic scenarios (as shown in Figure 5) are the following.

- BASELINE: During this phase, reference data from the driver are collected for 10 min. In particular, during this phase the driver is required to sit still inside the vehicle with the car engine turned *off*, with the resulting data recorded as “reference” and further compared with the data collected during the whole driving test.
- BELTWAY: The driver is asked to drive on a beltway for 5 min.
- CO-DRIVER: The driver is asked to drive on urban roads for 15 min. During the first and last 5 min intervals of this phase, road indications are given to the driver by a co-driver sitting in the back of the vehicle, and during the central 5 min interval, the co-driver provides stressful stimuli to the driver by simply talking to the driver and providing some *rude* comments to raise a stress reaction. Moreover, it should be highlighted that the co-driver is the same for all participants and is *unrelated* to all of them—in terms of familiarity—and the traffic condition during this phase was slow to heavy due to urban roads conditions.
- RECOVERY: During this phase, data are collected for 10 min more in order to check the driver’s response after a recovery phase. The driver is required to sit still inside the vehicle with the car engine turned *off* (as in the BASELINE phase). Finally, additional surveys about the perceived stress are also administered to investigate the impact of the driving test on the subject’s status.

**Figure 6:**
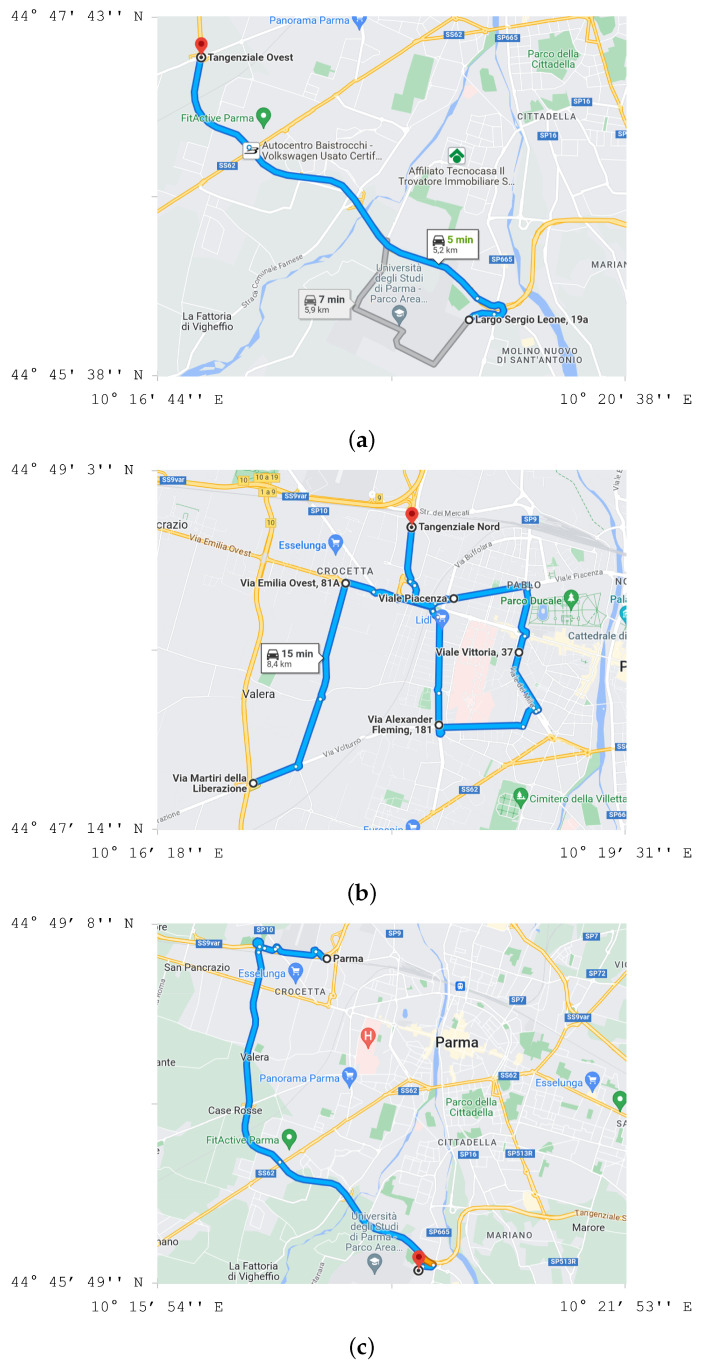
Beltway (**a**,**c**) and urban (**b**) roads crossed in the city of Parma, Italy, during driving tests.

The experimental route followed during the aforementioned driving tests lies in the city of Parma, Italy, as shown in Figure 6. The maps in Figure 6a,c refer to beltway routes, whereas the map in Figure 6b refers to an urban route. For all maps, the precise latitude and longitude coordinates are shown.

#### 3.2.2. Simulated Driving

**Figure 7:**
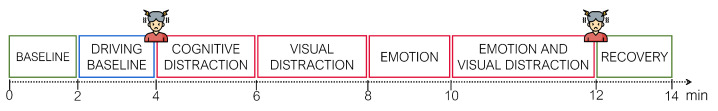
Driving protocol adopted in simulated scenarios.

The driving protocol defined for acquiring experimental data in a simulated environment (i.e., a driving simulator) is defined in [39], where the designed experimental scenario is described. In particular, during the simulated driving sessions, the participants are required to perform a series of distracting tasks in order to increase their level of perceived stress. For the sake of completeness, the various phases of a 14 min simulated driving test are shown in Figure 7, where, in detail, (i) reference data were acquired during the baseline phases and (ii) cognitive, visual and emotional distractions are emulated during the corresponding phases (as highlighted in Figure 7) in order to acquire data in stressful conditions. In particular, during the *first* cognitive distraction phase, the driver is asked to perform a memory exercise, named *n-back* (with n=2), where they have to listen to a sequence of letters read by an external voice and declare a match *if and only if* the last heard letter corresponds to the second-to-last heard letter. Then, the driver is asked to perform a *second* task to emulate both cognitive and visual distraction, during which they have to write on a cellphone a sequence of city names read by an external voice. During the *last* emotional distraction phase, the driver listens to an audio track and is asked to write on a cellphone the emotions that they are feeling. Finally, data at rest are collected during the recovery phase. It is finally noteworthy to highlight that none of the participants took part in both real and simulated driving scenarios, and that since cognitive, visual and emotional distractions are induced in the simulated driving tests, the type of stress in this specific scenario is considered both mental end emotional.

## 4. Data Analysis

In order to extract useful information from the data collected according to the protocols detailed in Section 3.2, different dedicated processing techniques are implemented. In particular, physiological and thermal data are processed separately in order to compute specific indices related to the stress status of the driver, as detailed and described in the following.

### 4.1. Physiological Data Analysis

Physiological indices of interest, i.e., HR and ECG signals, are acquired every 40 ms by the Equivital EQ02 Life Monitor sensor described in Section 2.1. Then, raw ECG signals are properly processed with the LabChartPro 5.0 software [40] to correctly detect R wave peaks—the R wave is an upward deflection in the ECG signal and represents early ventricular depolarization [41]. These data are of interest to evaluate the time interval between consecutive R peaks, also referred to as an R-R interval. This interval represents the time interval between normal heart beats, and its variations are expedient to quantifying the HRV, which is considered to evaluate the stress level. In particular, the Root Mean Square of Successive Difference (RMSSD, dim: [ms]) between consecutive R-R intervals is computed over a window of *N* samples, for each phase of the considered driving protocol, as follows:

(1) $$\mathrm{RMSSD}=\sqrt{\frac{\sum_{n=2}^{N}{\left({\mathrm{RR}}_{n}-{\mathrm{RR}}_{n-1}\right)}^{2}}{n-1}}$$

where ${\mathrm{RR}}_{n}$ represents the duration in ms of the *n*-th R-R interval, with n∈{2,…,N}, and the number of samples *N* is given by D/40, where *D* is the duration in ms of the considered phase and data are acquired every 40 ms. The RMSSD reflects the activity of the parasympathetic system, which is part of the ANS and regulates body functions at rest [42]. In particular, the vagus nerve is the main component of the parasympathetic system and is responsible for the heart rate reduction under relaxed conditions. On the other hand, the activity of the vagus nerve is reduced under stress conditions causing heart rate accelerations. Thus, reduced values of RMSSD are associated with a reduced activity of the vagus nerve as, for example, under higher levels of stress.

### 4.2. Thermal Data Analysis

**Figure 8:**
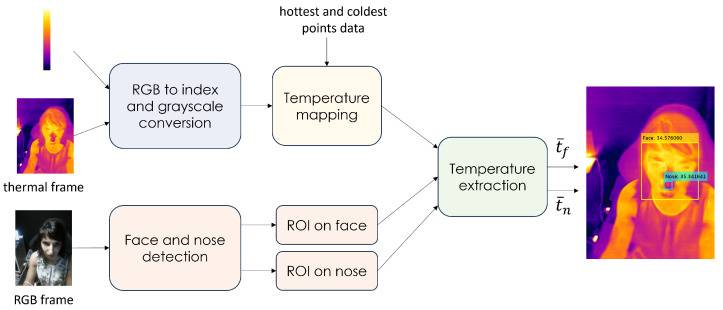
Dedicated algorithm for thermal data processing.

Thermal images and additional information of interest—namely, temperature ranges detected by the thermal camera and the pixel coordinates of the framed hottest and coldest points—were acquired by the FLIR One Pro Lt device described in Section 2.2 every 5 s and processed according to a dedicated algorithm, for which its block diagram is shown in Figure 8.

More in detail, the proposed algorithm retrieves the temperature information related to the considered thermal images, which are coded according to the RGB standard. Hence, a simple temperature mapping is performed in order to associate the RGB values of each pixel in a considered thermal image with their corresponding actual temperature values (dim: [°C]). For the sake of mapping, each thermal image, coded according to the RGB standard, is first indexed according to a specific color map, i.e., the temperature bar provided by the FLIR One Pro Lt device as additional information, in order to associate each RGB value within the image (representing the pixel intensity) to a unique integer index. This indexed image is then converted to grayscale to reduce the computational complexity of the mapping operation. The derived mapping rule is the following:

(2) $$I_{t}=t\left(l_{1},l_{2}\right)+\left[t\left(h_{1},h_{2}\right)-t\left(l_{1},l_{2}\right)\right]I_{i}$$

where $I_{t}$ and $I_{i}$ are two-dimensional matrices (dim: [pixel × pixel]) representing the thermal image with restored actual temperature values and the original RGB thermal image after being converted to a grayscale indexed image, respectively; $t(l_{1},l_{2})$ and $t(h_{1},h_{2})$ are the coldest and hottest temperature values (dim: [°C]) related to the pixels at position $\left(l_{1},l_{2}\right)$ and $\left(h_{1},h_{2}\right)$ within the indexed image $I_{i}$, respectively.

The RGB frames collected along with the thermal frames are also processed in order to extract two Regions Of Interest (ROIs) on the subject’s face. In particular, the MediaPipe Face Detector framework [43] is exploited to detect the face and nose of the driver in each RGB image. Then, two rectangular ROIs (centered at the nose and face) are extracted from the corresponding thermal image, and the mean temperatures are computed within the selected areas as follows:

(3) $$\bar{t}=\frac{1}{WH}\sum_{w=0}^{W-1}\sum_{h=0}^{H-1}t\left(w,h\right)$$

where *W* and *H* represent the width and height (in pixels) of the considered ROI, respectively, and t(w,h) is the temperature value associated with the pixel at position (w,h).

Finally, a check procedure is introduced in order to discard incorrectly detected ROIs. In detail, a nose ROI is ignored if the top-left corner $y_{0}^{\left(n-\mathrm{roi}\right)}\in \left\{0,\ldots ,H-1\right\}$ of the corresponding nasal area falls above 80% or below 25% of the height $h^{\left(f-\mathrm{roi}\right)}\in \left\{0,\ldots ,H-1\right\}$ of the face region, i.e., $y_{0}^{\left(n-\mathrm{roi}\right)}<0.25h^{\left(f-\mathrm{roi}\right)}$ or $y_{0}^{\left(n-\mathrm{roi}\right)}>0.8h^{\left(f-\mathrm{roi}\right)}$.

### 4.3. Arousal Extraction

Finally, in order to quantify the real-time psycho-physiological activation of the subject as a response to the driving task, the arousal index φ is obtained on the basis of the RMSSD value computed on the time interval corresponding to the current phase *C* (denoted as ${\mathrm{RMSSD}}_{C}$) with respect to the RMSSD value obtained on the time interval corresponding to the baseline phase (denoted as ${\mathrm{RMSSD}}_{B}$). A pseudo-code representation of the algorithm defined to compute the arousal φ is shown in Algorithm 1. In detail, with reference to Algorithm 1, *E* denotes the total number of driving phases in the considered protocol (excluding the baseline phase) and is set to 4 and 6 for real and simulated driving, respectively (as shown in Figure 5 and Figure 7); *s* represents the granularity of the arousal and is set to 0.1, in order to let the algorithm provide 10 different levels of arousal (namely: φ∈{0.1,0.2,0.3,…,1}); and $\Delta_{{\mathrm{RMSSD}}_{\mathrm{rat}}}^{\left(\min\right)}$ and $\Delta_{{\mathrm{RMSSD}}_{\mathrm{rat}}}^{\left(\max\right)}$ represent the minimum and maximum variation in the value $\Delta_{{\mathrm{RMSSD}}_{\mathrm{rat}}}$ that can be measured during the driving task, and they are set to 10 and 120, respectively, on the basis of an experimental values tuning phase.
**Algorithm 1** Pseudo-code of the arousal φ extraction.1:φ←0, e←12:s←granularityofthearousal3:$\Delta_{{\mathrm{RMSSD}}_{\mathrm{rat}}}^{\left(\min\right)}\leftarrow \mathrm{minimum}\;\mathrm{threshold}$4:$\Delta_{{\mathrm{RMSSD}}_{\mathrm{rat}}}^{\left(\max\right)}\leftarrow \mathrm{maximum}\;\mathrm{threshold}$5:${\mathrm{RMSSD}}_{B}\leftarrow \sqrt{\frac{\sum_{n=2}^{N_{B}}{\left({\mathrm{RR}}_{n}-{\mathrm{RR}}_{n-1}\right)}^{2}}{n-1}}$6:**while** e<E **do**7:   ${\mathrm{RMSSD}}_{C}\leftarrow \sqrt{\frac{\sum_{n=2}^{N_{C}}{\left({\mathrm{RR}}_{n}-{\mathrm{RR}}_{n-1}\right)}^{2}}{n-1}}$8:   $\Delta_{\mathrm{RMSSD}}\leftarrow {\mathrm{RMSSD}}_{B}-{\mathrm{RMSSD}}_{C}$9:   $\Delta_{{\mathrm{RMSSD}}_{\mathrm{rat}}}\leftarrow \mathrm{abs}\left(\frac{\Delta_{\mathrm{RMSSD}}\cdot 100}{{\mathrm{RMSSD}}_{B}}\right)$10:  **if** $\Delta_{\mathrm{RMSSD}}<0$ **then**11:     **if** $\Delta_{{\mathrm{RMSSD}}_{\mathrm{rat}}}<\Delta_{{\mathrm{RMSSD}}_{\mathrm{rat}}}^{\left(\min\right)}$ **then**12:        φ←013:     **else if** $\Delta_{{\mathrm{RMSSD}}_{\mathrm{rat}}}<\Delta_{{\mathrm{RMSSD}}_{\mathrm{rat}}}^{\left(\max\right)}$ **then**14:        $\varphi \leftarrow \frac{1}{10}\lceil \frac{\Delta_{{\mathrm{RMSSD}}_{\mathrm{rat}}}-\Delta_{{\mathrm{RMSSD}}_{\mathrm{rat}}}^{\left(\min\right)}}{s}\rceil$15:     **end if**16:   **else**17:     φ←018:   **end if**19:   e←e+120:**end while**

## 5. Experimental Results

In order to demonstrate the feasibility of the proposed IoT-oriented DMS, several driving sessions were performed both in simulated and realistic scenarios. To this end, the psycho-physiological data of interest were collected according to the acquisition protocols described in Section 3.2 and processed according to the algorithms detailed in Section 4. More in detail, the driving tests were administered to 28 healthy subjects (13 women and 15 men) and 40 healthy subjects (20 women and 20 men), aged 20 to 50, in the case of simulated and realistic scenarios, respectively. Each participant was required to sign an informed consent, to hold a driving license from at least 3 years and to own a car (which was used as the mobile vehicle during their driving session and equipped as shown in Figure 1).

### 5.1. Simulated Scenarios

**Figure 9:**
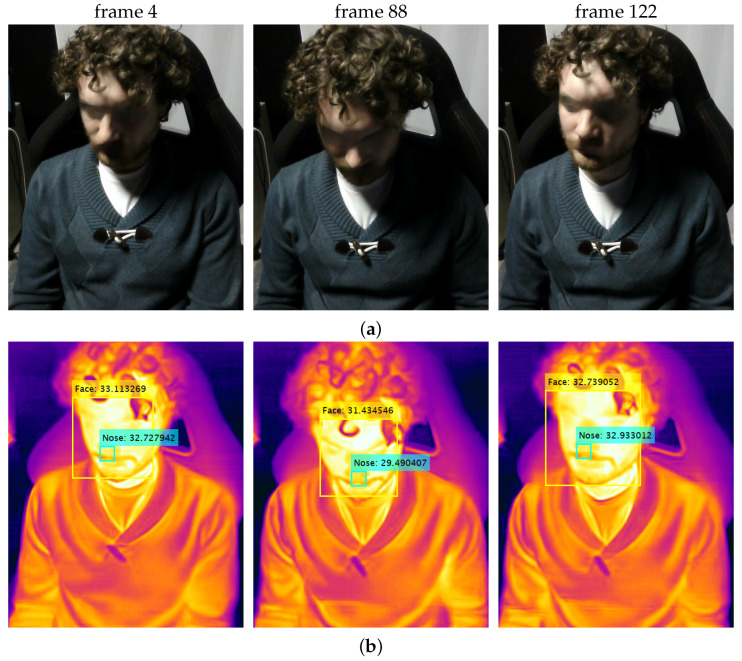
Samples of (**a**) original RGB and (**b**) processed thermal frames extracted during driving session $S_{1}$.

**Figure 10:**
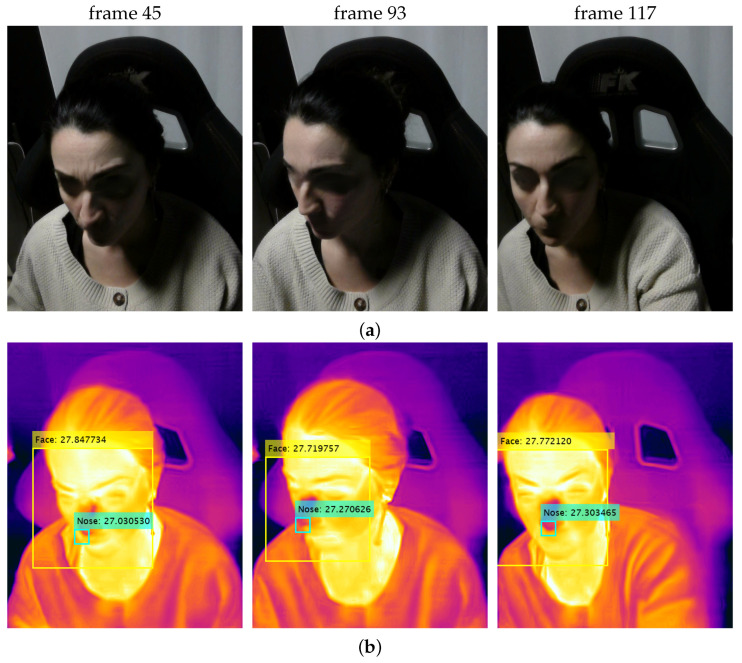
Samples of (**a**) original RGB and (**b**) processed thermal frames extracted during driving session $S_{2}$.

The results of two simulated driving sessions, denoted as $S_{1}$ and $S_{2}$, are detailed hereafter, with examples of RGB and thermal frames extracted from the corresponding video sequences by the FLIR One Pro Lt sensor and properly processed by the algorithm described in Section 4.2, as shown in Figure 9 and Figure 10, respectively.

**Figure 11:**
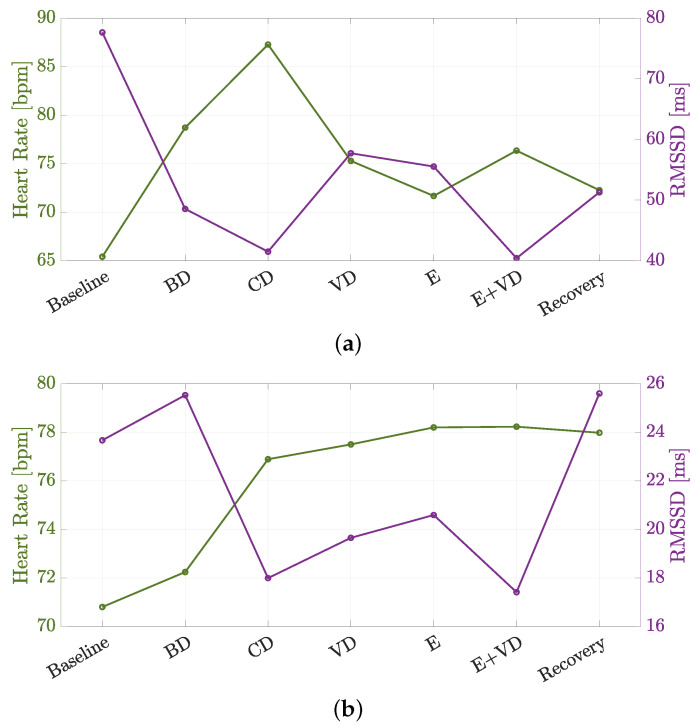
Physiological data extracted during (**a**) $S_{1}$ and (**b**) $S_{2}$.

The physiological data of interest collected during $S_{1}$ and $S_{2}$ are shown in Figure 11a and Figure 11b, respectively. In particular, the mean HR and RMSSD are shown for each phase of the driving protocol detailed in Section 3.2 (and depicted in Figure 7). To this end, the RMSSD values are computed according to Equation (1) with N=D/40=3000, considering that each phase has a duration of 2 min (namely, D=1200 ms) and data have been acquired every 40 ms. The various phases are labeled as Baseline, Baseline Driving (BD), Cognitive Distraction (CD), Visual Distraction (VD), Emotion (E), Emotion and Visual Distraction (E+VD), and Recovery.

Therefore, through carefully analyzing the results shown in Figure 11a,b, a psycho-physiological activation of the drivers can be observed during both sessions. In detail, the RMSSD values acquired during the epochs of the protocol corresponding to stressful conditions (i.e., CD, VD, E, E + VD) are indeed lower than the baseline values acquired during Baseline and BD epochs.

Considering Figure 11a, the most significant activation of the subject is observed during the CD phase, where a very small value of RMSSD (namely, 41.49 ms) is computed, and a very high value of mean HR (namely, 87.27 bpm) is recorded, indicating a reduced activity of the vagus nerve, as typically observed in stressful circumstances. A similar condition is observed during the E+VD phase, where the value of the RMSSD index is 40.41 ms, and the mean HR is 76.34 bpm. Likewise, considering Figure 11b, the subject is mainly activated during the CD and E+VD phases, where the values of the RMSSD are 17.99 ms and 17.41 ms, respectively, while the values of the mean HR are 76.89 bpm and 78.23 bpm, respectively.

Moreover, it can be observed that during the final Recovery phase (in both Figure 11a,b), RMSSD increases, and the mean HR decreases with respect to the values obtained during the previous E+VD phase, indicating that both subjects are experiencing a relaxed condition once the driving session is over.

**Figure 12:**
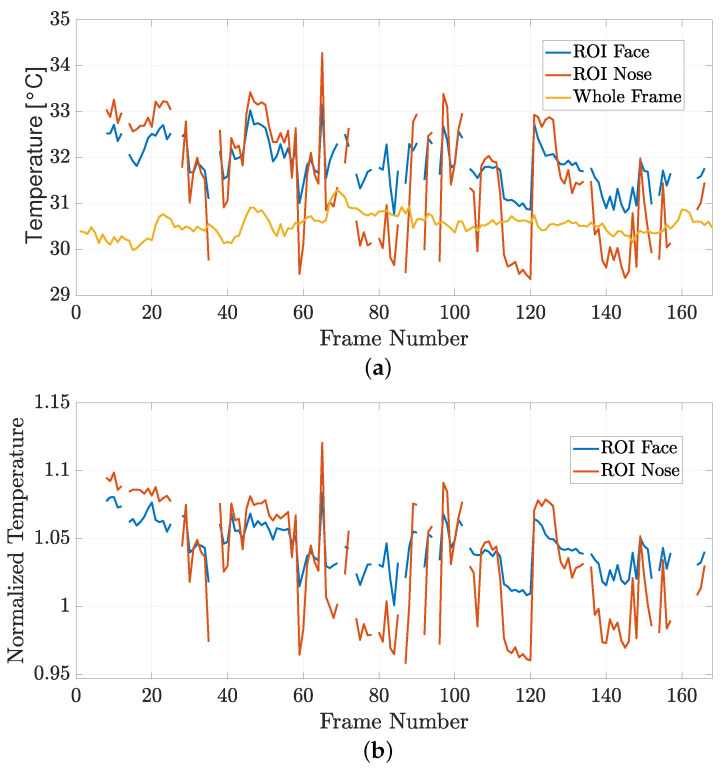
(**a**) Mean temperatures and (**b**) normalized mean temperatures extracted during $S_{1}$.

**Figure 13:**
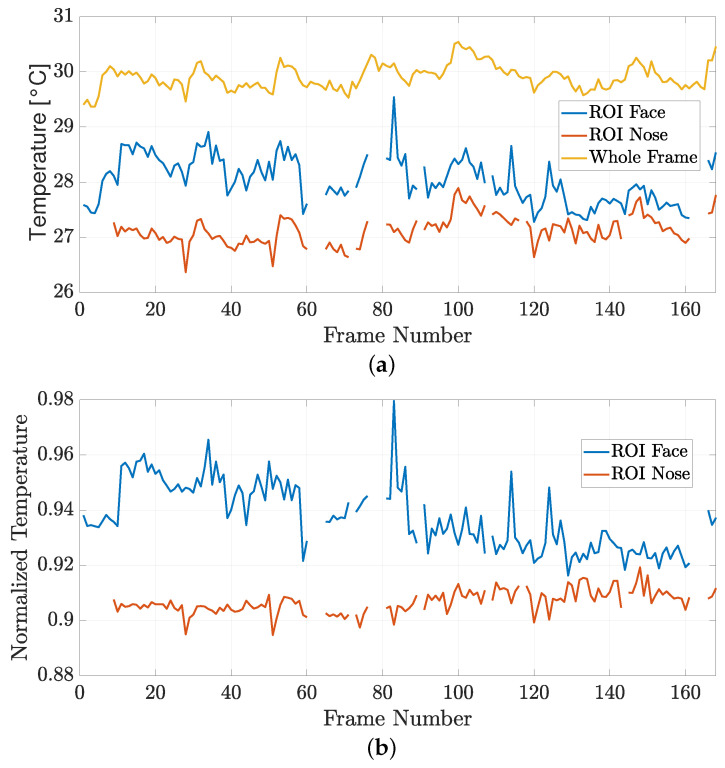
(**a**) Mean temperatures and (**b**) normalized mean temperatures extracted during $S_{2}$.

As a further analysis, the mean temperatures extracted from the thermal frames acquired during $S_{1}$ and $S_{2}$ are shown in Figure 12 and Figure 13, respectively. In particular, the total number of analyzed frames was equal to 168 for both sessions, corresponding to a 14 min total duration of the simulated driving protocol, considering that thermal data are acquired every 5 s.

For Figure 12a and Figure 13a, the mean temperature values (dim: [°C]) were computed by applying Equation (3) on the drivers’ face and nose ROIs reported for each acquired frame, along with the mean temperature extracted from the whole frame (detected and extracted through the processing performed by means of the MediaPipe Face Detector framework). However, at some time instants, artifacts, possibly due to movements of the subjects, may lead to incorrect detections, as visible in Figure 12 and Figure 13, at those frame indices where the temperature curves are not defined.

In order to neutralize the effects of environmental factors, e.g., air conditioning and/or heating inside the vehicle’s cabin, the mean temperature values extracted from face and nose ROIs are also normalized, for each acquired frame, with respect to the mean temperature obtained over the whole frame. In fact, this normalization allows us to compensate the effects of undesired temperature oscillations inside the vehicle’s cabin, which are unrelated to the stress perceived by the drivers. To this end, the normalized temperature curves associated with $S_{1}$ and $S_{2}$ are shown in Figure 12b and Figure 13b, respectively.

**Figure 14:**
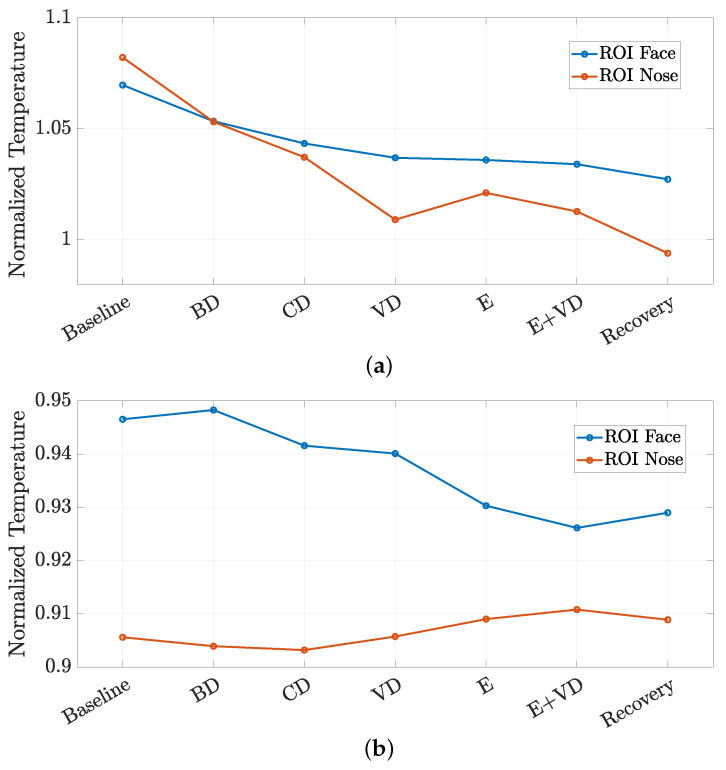
Mean temperatures averaged over time windows corresponding to the epochs of the driving protocol for (**a**) $S_{1}$ and (**b**) $S_{2}$.

In order to fairly compare physiological and thermal data, the normalized mean temperatures shown in Figure 12b and Figure 13b were temporally averaged over each phase of the simulated driving protocol, lasting 2 min, obtaining the normalized temporally averaged temperature shown in Figure 14a and Figure 14b, associated with $S_{1}$ and $S_{2}$, respectively.

In observing these results, it can be noticed the decreasing trend in the temperature curves in Figure 14a, which suggests a potential stress-related alteration in the psycho-physiological status of the driver during $S_{1}$. The same conclusion may be drawn by observing the temperature curve related to the face ROI shown in Figure 14b. On the basis of these observations, it can be claimed that the temperature data are in agreement with the physiological data highlighted in Figure 11. Indeed, from CD to E+VD phases, the temperature curves are descending, whereas the RMSSD curves are ascending: both trends indicate a psycho-psychological activation of the subject during the phases associated with stressful conditions. On the other hand, the temperature curve related to the nose ROI shown in Figure 14b exhibits a slightly increasing trend from the CD to E phases that may be due to partially incorrect ROI detection. However, a deeper analysis of the thermal data is yet under consideration.

**Figure 15:**
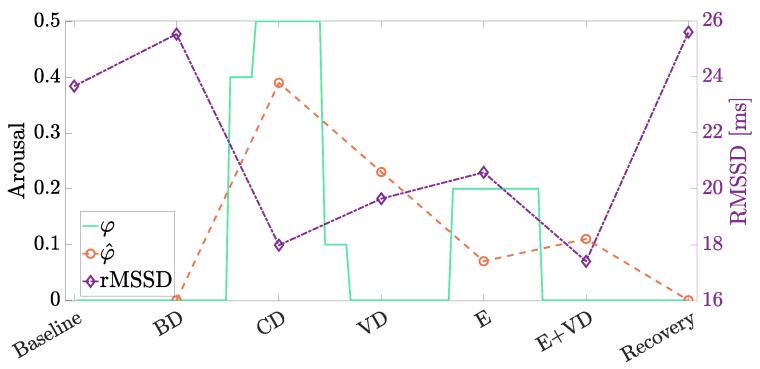
Experimental RMSSD (purple), experimental arousal (green, calculated each 5 s) and mean arousal (orange, calculated over each epoch) for scenario $S_{2}$.

Finally, in order to further strengthen the agreement between the experimental physiological data and arousal φ calculated on the basis of Algorithm 1, in Figure 15, the trends related to session $S_{2}$ are shown.

In detail, with reference to Figure 15, there is a clear interest in evaluating the mean arousal $\hat{\varphi }$, computed as the arithmetic average of the arousal values φ (obtained each 5 s on the basis of Algorithm 1) over each phase of the driving protocol. It can be observed that $\hat{\varphi }$ shows a clear “inverse” behavior with respect to the RMSSD processed over each phase, thus confirming how the arousal estimation might be effective in these scenarios. Finally, it should be mentioned that the current version of the arousal algorithm only considers HRV data, whereas the integration of thermal data is yet under investigation. In this first experimental analysis, we limited the investigation to the IoT-based system behavior evaluated in terms of thermal data acquisition from specific regions of interest (namely, face and nose).

### 5.2. Real Scenarios

**Figure 16:**
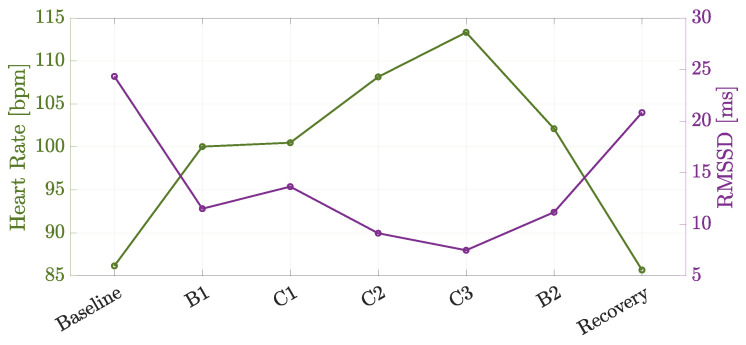
Physiological data extracted during a real driving session.

Finally, the results of a driving session run in a realistic scenario, in terms of collected physiological data, are shown in Figure 16. In particular, the mean HR and RMSSD values have been computed according to Equation (1) with N=7500, considering phases with a fixed duration of 5 min and data acquired every 40 ms. To this purpose, the last 5 min of the Baseline and Recovery phase recordings are taken into account for the physiological data analysis described in Section 4.1. The obtained mean HR and RMSSD values are plotted in Figure 16 for each phase of the considered protocol. In detail, the different phases were labeled as Baseline, Beltway 1 (B1), Co-Driver 1 (C1), Co-Driver 2 (C2), Co-Driver 3 (C3), Beltway 2 (B2) and Recovery, where C1, C2 and C3 correspond to the CO-DRIVER phases detailed in Section 3.2.1—in fact, during C1 and C3, only road indications are given to the driver, whereas during C3 phase, stressful stimuli are also induced.

Hence, it can be observed that in stressful conditions, i.e., from phases C1 to C3 in Figure 16, the driver is subject to a psycho-physiological activation, with the mean HR exhibiting an increasing trend. At the opposite, RMSSD decreases, suggesting a reduction in the vagus nerve activity and an increase in the perceived stress.

Since the feasibility of the proposed system in real scenarios is a much more difficult to achieve compared to in controlled environments, i.e., the simulator, it was not possible to exploit the thermal camera during this driving session. The installation of the thermal camera is, indeed, highly sensitive to road and vehicle conditions, which may cause vibrations, possibly interfering with its correct functioning. Moreover, the vehicle cabin is more likely to be subject to temperature variations, which may be hardly detectable, unless more sensors, such as thermometers, are installed. Hence, thermal data are not reported for this illustrative example.

## 6. Conclusions and Future Activities

In this work, an IoT-based monitoring system that assesses the psycho-physiological status of a driver was developed and presented. Heterogeneous sensors, i.e., a wearable bodice and a thermal camera, are integrated in the proposed architecture and used to sense various physiological indices of interest, including HR, ECG signals and skin temperature. Data are collected according to well-defined protocols and properly processed by dedicated algorithms, specifically implemented for this purpose. In particular, the HRV of the driver is evaluated by computing the RMSSD index, which reflects the activity of the parasympathetic system and is considered a physiological index of stress. Variations in the skin temperature on the subject’s face and nose regions are also analyzed, since they are associated with stress-related physiological activation. Finally, experimental results were obtained in both simulated and real driving scenarios, and analyzed in order to demonstrate the relationship between the driving task and increased levels of perceived stress. The feasibility of the system was also demonstrated.

As future activities, the extraction of vehicular data from embedded inertial sensors and on-board units (OBUs) could be considered part of the proposed JDVS module. Also, the correlation between vehicular data and physiological parameters could be further investigated in order to evaluate the relationship between the driving behavior and the detected level of stress. Moreover, an optimization of the whole system could be foreseen by better integrating the thermal camera sensor in realistic driving scenarios. Finally, the use of different devices could be investigated, such as a smartwatch used to extract physiological measurements in a less-invasive way.

## Data Availability

Data are contained within the article.

## Abbreviations

The following abbreviations are used in this manuscript:

ANS	Autonomic Nervous System;
AP	Access Point;
BLE	Bluetooth Low Energy;
CAN	Controller Area Network;
DAS	Driver Assistance System;
DMS	Driver Monitoring System;
ECG	ElectroCardioGram;
ECU	Engine Control Unit;
HR	Heart Rate;
HRV	Heart Rate Variability;
IoT	Internet of Things;
JDVS	Joint Driver–Vehicle Status;
ML	Machine Learning;
MQTT	Message Queuing Telemetry Transport;
OBU	On-Board Unit;
RMSSD	Root Mean Square of Successive Difference;
ROI	Region Of Interest;
RR	Respiratory Rate;
SEM	Sensor Electronics Module;
TCP	Transmission Control Protocol.
